# Bacterial metallothionein, PmtA, a novel stress protein found on the bacterial surface of *Pseudomonas aeruginosa* and involved in management of oxidative stress and phagocytosis

**DOI:** 10.1128/msphere.00210-24

**Published:** 2024-05-07

**Authors:** Michele Maltz-Matyschsyk, Clare K. Melchiorre, David A. Knecht, Michael A. Lynes

**Affiliations:** 1 Department of Molecular and Cell Biology, University of Connecticut, Storrs, Connecticut, USA; The University of Arizona, Tucson, Arizona, USA

**Keywords:** *Pseudomonas aeruginosa*, bacterial metallothionein, innate immune system, phagocytosis, oxidative stress

## Abstract

Metallothioneins (MTs) are small cysteine-rich proteins that play important roles in homeostasis and protection against heavy metal toxicity and oxidative stress. The opportunistic pathogen, *Pseudomonas aeruginosa,* expresses a bacterial MT known as PmtA. Utilizing genetically modified *P. aeruginosa* PAO1 strains (a human clinical wound isolate), we show that inducing *pmtA* increases levels of pyocyanin and biofilm compared to other PAO1 isogenic strains, supporting previous results that *pmtA* is important for pyocyanin and biofilm production. We also show that overexpression of *pmtA in vitro* provides protection for cells exposed to oxidants, which is a characteristic of inflammation, indicating a role for PmtA as an antioxidant in inflammation. We found that a *pmtA* clean deletion mutant is phagocytized faster than other PAO1 isogenic strains in THP-1 human macrophage cells, indicating that PmtA provides protection from the phagocytic attack. Interestingly, we observed that monoclonal anti-PmtA antibody binds to PmtA, which is accessible on the surface of PAO1 strains using both flow cytometry and enzyme-linked immunosorbent assay techniques. Finally, we investigated intracellular persistence of these PAO1 strains within THP-1 macrophages cells and found that the phagocytic endurance of PAO1 strains is affected by *pmtA* expression. These data show for the first time that a bacterial MT (*pmtA*) can play a role in the phagocytic process and can be found on the outer surface of PAO1. Our results suggest that PmtA plays a role both in protection from oxidative stress and in the resistance to the host’s innate immune response, identifying PmtA as a potential therapeutic target in *P. aeruginosa* infection.

**IMPORTANCE:**

The pathogen *Pseudomonas aeruginosa* is a highly problematic multidrug-resistant (MDR) pathogen with complex virulence networks. MDR *P. aeruginosa* infections have been associated with increased clinical visits, very poor healthcare outcomes, and these infections are ranked as critical on priority lists of both the Centers for Disease Control and Prevention and the World Health Organization. Known P. aeruginosa virulence factors have been extensively studied and are implicated in counteracting host defenses, causing direct damage to the host tissues, and increased microbial competitiveness. Targeting virulence factors has emerged as a new line of defense in the battle against MDR *P. aeruginosa* strains. Bacterial metallothionein is a newly recognized virulence factor that enables evasion of the host immune response. The studies described here identify mechanisms in which bacterial metallothionein (PmtA) plays a part in *P. aeruginosa* pathogenicity and identifies PmtA as a potential therapeutic target.

## INTRODUCTION

*Pseudomonas aeruginosa* is a non-fermenting, Gram-negative, opportunistic pathogen that ranks among the top five healthcare-associated infections nationwide (1, 2). The large and diverse arsenal of virulence factors found within *P. aeruginosa* make for a versatile pathogen that can effectively respond to immunological stressors (3–5). Most commonly, these bacteria are found in patients that are immunocompromised, have undergone invasive surgery, or who have underlying conditions such as diabetes or cystic fibrosis (6). *P. aeruginosa* infections also frequently arise with the use of medical devices such as ventilators, central lines, urinary catheters, and/or surgical/transplantation (4) and have been implicated as the causative agent of a wide variety of infection types (e.g., pneumonia, sepsis, keratitis, skin, bone, joint, endocarditis, and meningitis). *P. aeruginosa* infections associated with chronic lung disease, such as cystic fibrosis, ventilator-associated pneumonia (VAP), and chronic obstructive pulmonary disease, are persistent and often linked to an increase in morbidity and mortality (4). Coronavirus disease 2019 patients who develop VAP can be coinfected with several *Enterococcus faecium*, *Staphylococcus aureus*, *Klebsiella pneumoniae*, *Acinetobacter baumannii*, *Pseudomonas aeruginosa*, and *Enterobacter* species (ESKAPE) pathogens, including *Escherichia coli*, *K. pneumoniae*, *A. baumannii*, *S. aureus*, and *P. aeruginosa* (7). Furthermore, severe acute respiratory syndrome coronavirus 2 infection provides a lung environment that is conducive to rapid adaptive evolution of chronic *P. aeruginosa* infections (8). In burn wounds, *P. aeruginosa* is prevalent in 59% of patients with extensive burns and exacerbates morbidity and mortality in these patients as well (9). Quiet often, these infections are recurrent as a consequence of *P. aeruginosa’s* ability to develop a population of persister cells (slow or nondividing cells) within biofilms under environmental stressors such as oxidative stress, nutrient deprivation, and antibiotic treatments (10).

*P. aeruginosa* expresses a small-molecular-weight (approximately 9 kDa), cysteine-rich protein (PmtA) identified as a member of the stress-response family of metallothionein (MT) proteins (11, 12). Recent bioinformatic analysis of the *Pseudomonas* genome database has shown that bacterial MTs are highly conserved in 90% of *Pseudomonas* species (13). Comparative genomics of PmtA protein sequences has also revealed close orthologous relationships between four of the six ESKAPE pathogens (*K. pneumoniae*, *A. baumannii*, *P. aeruginosa*, and *Enterobacter* species), all of which share motifs and interacting residues (14). These bacteria are also multidrug resistant and found in similar infection sites such as the lungs, blood, urinary tract, and skin (15, 16). MT family proteins have been well characterized in eukaryotes as essential for zinc and copper homeostasis, protection against oxidative stress, and for their ability to modify a variety of immune activities (11, 12). Bacterial MTs share sequence homology, antioxidant chemistry, and heavy metal-binding capacity with eukaryotic MT, but the impact of bacterial MTs on virulence and infection have not been thoroughly examined. While studies have shown bacterial MT involvement in metal-binding function (17–19), our studies with PAO1 have found no evidence for PmtA involvement in detoxification of either zinc, an essential divalent heavy metal cation, or cadmium, a divalent heavy metal toxicant (20). *P. aeruginosa* can express many other metal-binding proteins that could easily compensate for the lack of PmtA in our deletion mutant; this compensation could be the reason for this absence of evidence (21). We recently have shown that *P. aeruginosa* metallothionein (PmtA) plays a role in the expression of pyocyanin and in the formation of biofilms in *P. aeruginosa*. Within that study, we also showed that decreased PmtA expression is associated with decreased virulence in the waxworm (*Galleria mellonella*), a non-mammalian model of innate immune response to infection, and that a clean deletion mutant (Δ*pmtA*) displays increased susceptibility to the antibiotics that are currently used to treat *P. aeruginosa* infection (20).

In a recently published study comparing *P. aeruginosa* genes expressed in log-phage cultures to genes expressed during the colonization of a skin wound, *pmtA* (Locus tag: PA2140) was shown to be upregulated between 5 and 9 days post colonization, indicating a role for *pmtA* in progression of disease (22). PmtA has also been shown to be important for both burns and chronic surgical wound infections (23). These skin infections and the associated tissue damage elicit an immediate and robust innate immune response, including infiltration of immune cells like macrophages and neutrophils that produce oxygen-free radicals used to destroy the pathogens during phagocytosis (24). The survival of pathogens under oxidative stress depends on the ability to evade the host response by either detoxifying the reactive oxygen species (ROS) environment or escaping phagocytic engulfment.

Here, we investigate the role of *P. aeruginosa* PmtA in the innate immune response. Using a clean deletion *pmtA* mutant (previously characterized [20]) and a chromosomal-overexpressor *pmtA* mutant of PAO1 (P_BAD_*pmtA*) driven by an arabinose-inducible promoter, we examine PmtAs’ importance in enhancing bacterial survival when encountering a host’s innate immune response.

## RESULTS

### Characterization of overexpression of *pmtA* in PAO1

**Fig 1:**
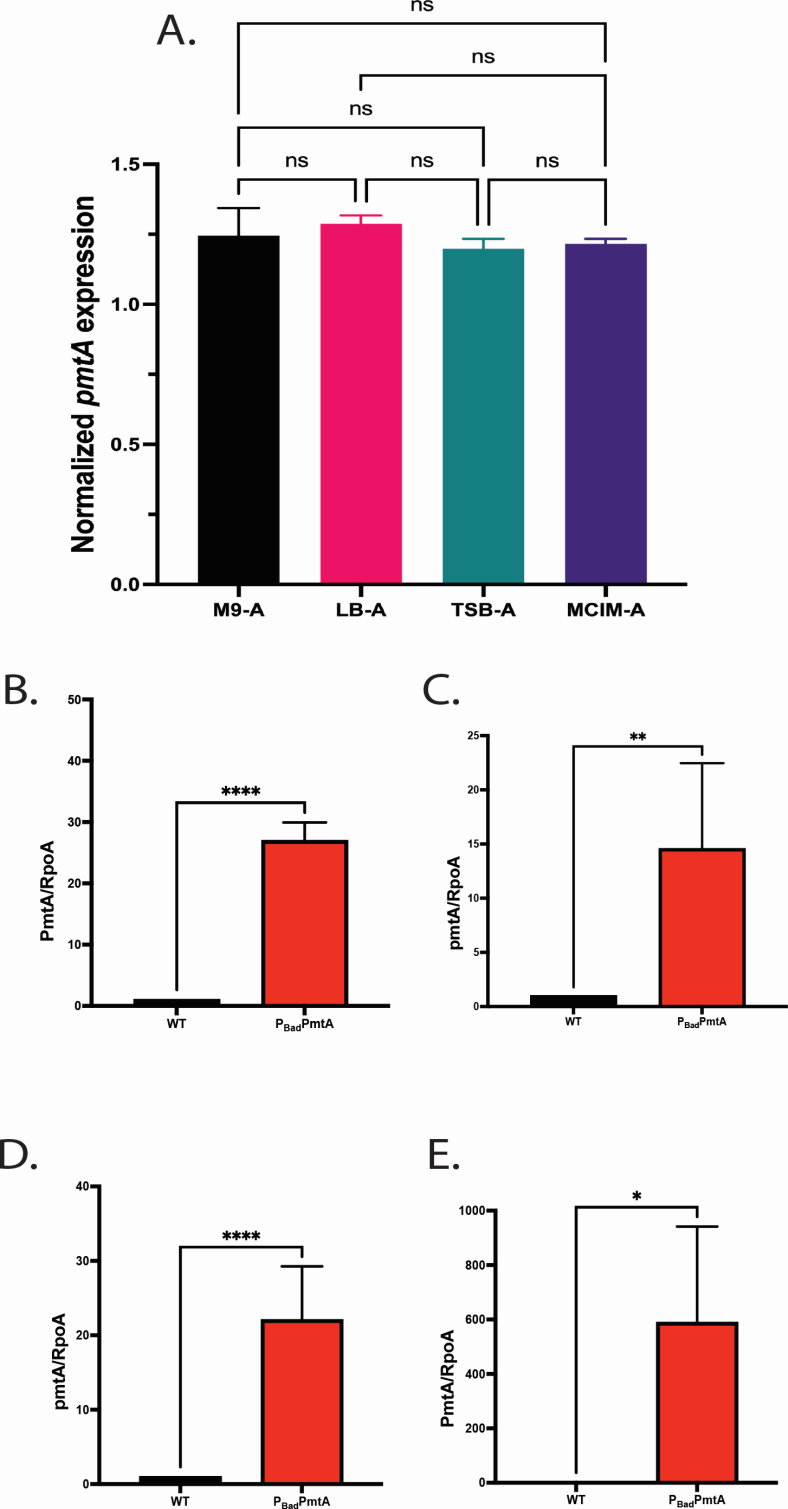
Wild-type PAO1 *pmtA* expression in different media and validation of P_BAD_*pmtA* promotor activation. (**A**) Nutritional effects on *pmtA* gene expression in WT PAO1 grown in different media in the presence of arabinose showed no differences when normalized to RNA polymerase, subunit alpha (RpoA). One-way analysis of variance (ANOVA) analysis was performed (*P < 0.05, **P < 0.01, ***P < 0.001, ****P < 0.0001; ns, not significant). (**B–E**) Activation of PAO1 strain P_BAD_*pmtA*; P_BAD_ promotor-driven overexpression of *pmtA* was tested in different growth media used in this study; M9 media (**B**), LB (**C**), TSB (**D**), and infection media (**E**) all supplemented with arabinose. Total RNA was extracted from cells at 24 h and converted to cDNA. qPCR was performed on a CFX96 Real-Time Thermocycler, and relative gene expression values for *pmtA* were calculated using the cycle threshold value compared to the RNA polymerase, subunit alpha. The expression of *pmtA* in P_BAD_*PmtA* was significantly increased when compared to WT when arabinose is present in all media types indicating induction of *pmtA* on the inserted Tn7. An unpaired *t*-test analysis was performed (*P < 0.05, **P < 0.01, ***P < 0.001, ****P < 0.0001). The data are presented as the average of three biological replicates (± standard error of the mean) and are representative of three separate experiments.

**Fig 2:**
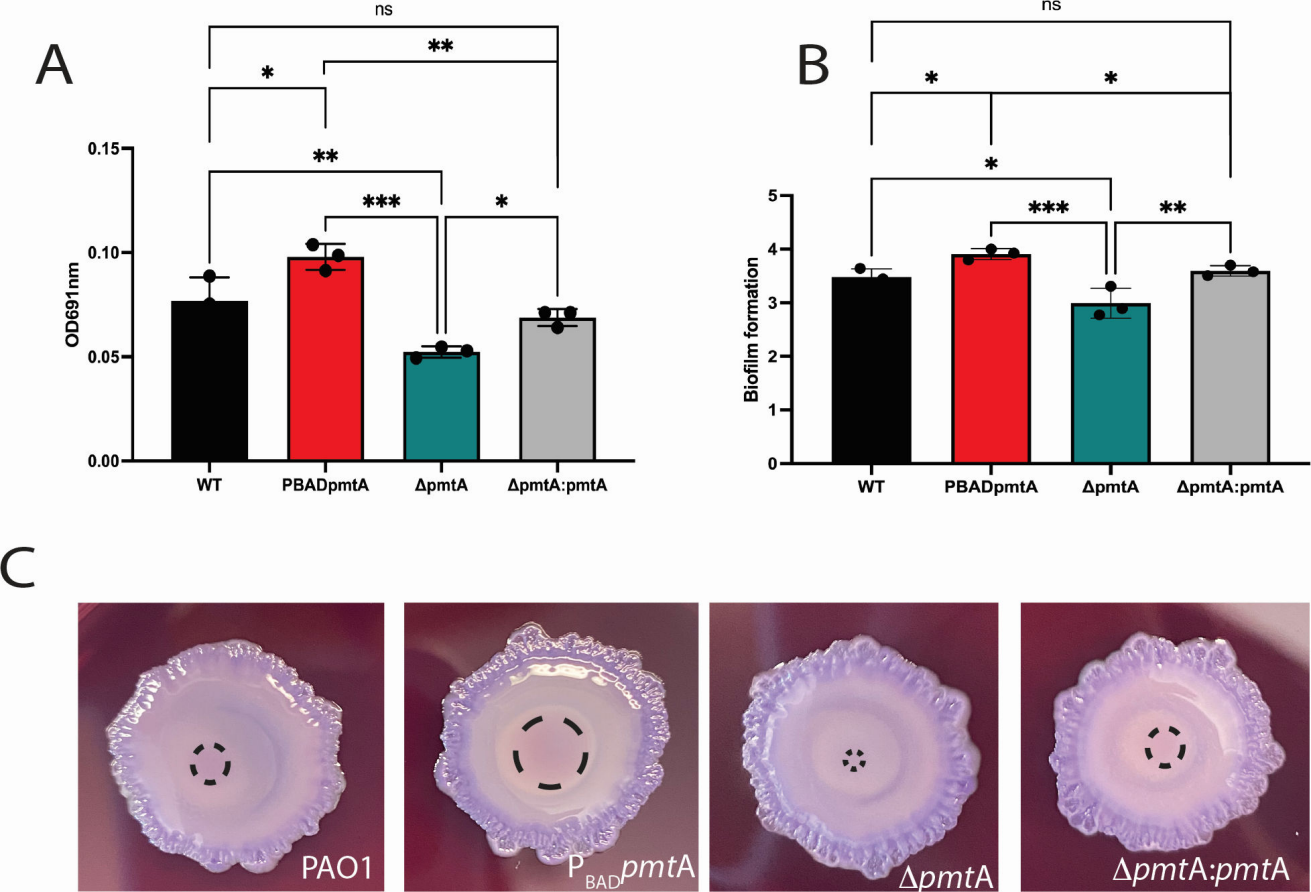
*In vitro* phenotypes from inducing overexpression of PmtA in *P. aeruginosa* with 1% arabinose. (**A**) Pyocyanin was measured at optical density at 691 nm (OD_691_). P_BAD_pmta shows increase in pyocyanin production in P_BAD_pmtA when compared to other PAO1 strains cultured in 1% arabinose. (**B**) Biofilms were stained with crystal violet, measured at OD_595_, and relative biofilm formation was calculated (20). Increased biofilm formation in P_BAD_pmtA was observed when compared to other PAO1 strains cultured in 1% arabinose. (**C**) Colony morphology on Congo red plates. The pink center (outlined by black dashes) indicates biofilm formation; P_BAD_pmtA has the largest extent of this stained center. Data are presented as the average of three replicates from individual colonies (± standard error of the mean), and the results presented are representative of three independent experiments. One-way ANOVA analysis was performed (*P < 0.05, **P < 0.01, ***P < 0.001, ****P < 0.0001) and compared to ΔpmtA. An unpaired *t*-test analysis was performed on WT and P_BAD_ pmtA; ΔpmtA:pmtA and P_BAD_ pmtA; WT and ΔpmtA:pmtA individually (*P < 0.05, **P < 0.01, ***P < 0.001, ****P < 0.0001, ns, not significant).

Overexpression of a wild-type gene can be a powerful tool to identify mutant phenotypes that may be missed by observing phenotypes associated with a clean deletion (25). We constructed a *pmtA*-overexpressing mutant (P_BAD_*pmtA*) using a broad-host range Tn7 transposon with a P_BAD_ arabinose-inducible promoter, pTJ19 (26) (Fig. S1). This P_BAD_ Tn7 contains the promoter of the arabinose operon and its regulatory gene, *araC*, allowing for tight control of expression (27). We inserted *pmtA* downstream of this promoter using EcoRI and HindIII restriction sites and the Gibson Assembly protocol. We verified the gene was in the correct orientation by sequencing and then conjugated this suicide delivery vector (pBADpmtA) into PAO1 (WT) generating *P_BAD_pmtA* (Fig. S1A). The insertion of the Tn7 into the chromosome was verified by PCR amplification of the *glmS* region, allowing for less concern for vector plasmid maintenance and burden within bacterial cells (Fig. S1B) (20, 26, 28, 29). No growth rate changes were observed in P_BAD_*pmtA* when grown in tryptic soy broth (TSB), Luria–Bertani (LB) (supplemented various levels of arabinose) or M9-A (arabinose as a sole carbon source) media when compared to either the WT or to other PAO1 isogenic strains (Table 1; Fig. S2 to S4). Using quantitative PCR (qPCR), we tested the effects of different media (i.e., TSB plus 1% arabinose, TSB-A; LB plus 1% arabinose, LB-A; M9-A; and mammalian cell infection media plus 1% arabinose [MCIM-A]) on the expression of *pmtA* in WT PAO1 alone and found that the amount of *pmtA* expressed did not change with nutrient availability (Fig. 1A). We next tested promotor activation in P_BAD_*pmtA* grown in M9-A, LB-A, TSB-A, or MCIM-A with arabinose and found that in the presence of arabinose, P_BAD_*pmtA* showed a 15- to 30-fold increase in *pmtA* expression when compared to wild type (Fig. 1B through E). The MCIM-A had the highest fold change difference in expression of *pmtA* when compared to WT: this could be due to the presence of fetal bovine serum (FBS) present in this media. Arabinose is the second most abundant pentose in plants and a component of cattle feed as such, which may represent an additional source of arabinose in the MCIM-A (30). Alternatively, the pBAD promoter has been shown to be regulated by virulence factor regulator (Vfr) which could be activated in the presences of serum (31). Since we have previously shown that the deletion of *pmtA* in PAO1 results in diminished pyocyanin production and biofilm formation, we investigated these same phenotypes in P_BAD_*pmtA* (Fig. 2). Inducing overexpression of *pmtA* in TSB 1% arabinose leads to more pyocyanin production (Fig. 2A) and greater biofilm formation (Fig. 2B and C) when compared to similarly treated WT and other isogenic PAO1 strains (Table 1). Interestingly, the overproduction of pyocyanin in P_BAD_*pmtA* does not lead to a decrease in the growth rate when compared to WT as has been seen in other studies where pyocyanin was induced (Fig. S2), suggesting that PmtA can also provide endogenous protection from pyocyanin-induced oxidative stress (32).

**TABLE 1:** Bacterial strains and plasmids used in the present study

Bacterial strain or plasmid	Characteristics	Source or reference
*Escherichia coli* strain		
NEB 5-alpha	General cloning strain	NEB
*Pseudomonas aeruginosa s*trains		
PAO1	Wild type (WT)	Manoil Lab UW
∆*pmtA*	PAO1∆PA2140	(20)
∆*pmtA*:*pmtA*	PAO1, ∆PA2140, miniTn7-Gm-*pmtA* Gent^R^	(20)
P_BAD_*pmtA*	PAO1, miniTn7-Tp-araC-P_BAD_*pmtA*, Tp^R^	This study
PAO1 *attTn7::dTomato*	miniTn7-Tp-araC-P_BAD_, constitutively expresses dTomato; Tp^R^	This study
∆*pmtA attTn7::dTomato*	PAO1∆PA2140, miniTn7-Tp-araC-P_BAD_, constitutively expresses dTomato; Tp^R^	This study
P_BAD_*pmtA* attTn7::sfGFP	PAO1, miniTn7-Tp-araC-P_BAD_*pmtA,* constitutively expresses sfGFP, Tp^R^	This study
Plasmids		
pTNS3	Helper plasmid encoding transposase gene necessary for the chromosomal integration of mini-Tn*7*	(33)
pRK2013	Helper plasmid for conjugation	(34)
pTJ1	Suicide delivery vector: JX559783; pUC18T-miniTn7-Tp-araC-P_BAD_-MCS, Tp^R^	(26)
pBADPmtA	Suicide delivery plasmid: pTJ1; miniTn7-Tp-araC-P_BAD_*pmtA*, Tp^R^	This study
pTW415	pTn7xKS with sfGFP expression scaffold; Amp^R^, Gent^R^	(35)
pTW416	pTn7xKS with dTomato expression scaffold; Amp^R^, Gent^R^	(35)
pPBADpmtAGFP	Suicide delivery vector: pTJ1; miniTn7-Tp-araC-P_BAD_*pmtA*, sfGFP expression scaffold; Tp^R^	This study
pdTomatoPBADTPTn7	Suicide delivery vector: pTJ1; miniTn7-Tp-araC-P_BAD_, with dTomato expression scaffold; Tp^R^	This study

### Overexpression of PmtA confers resistance to oxidative stress

**Fig 3:**
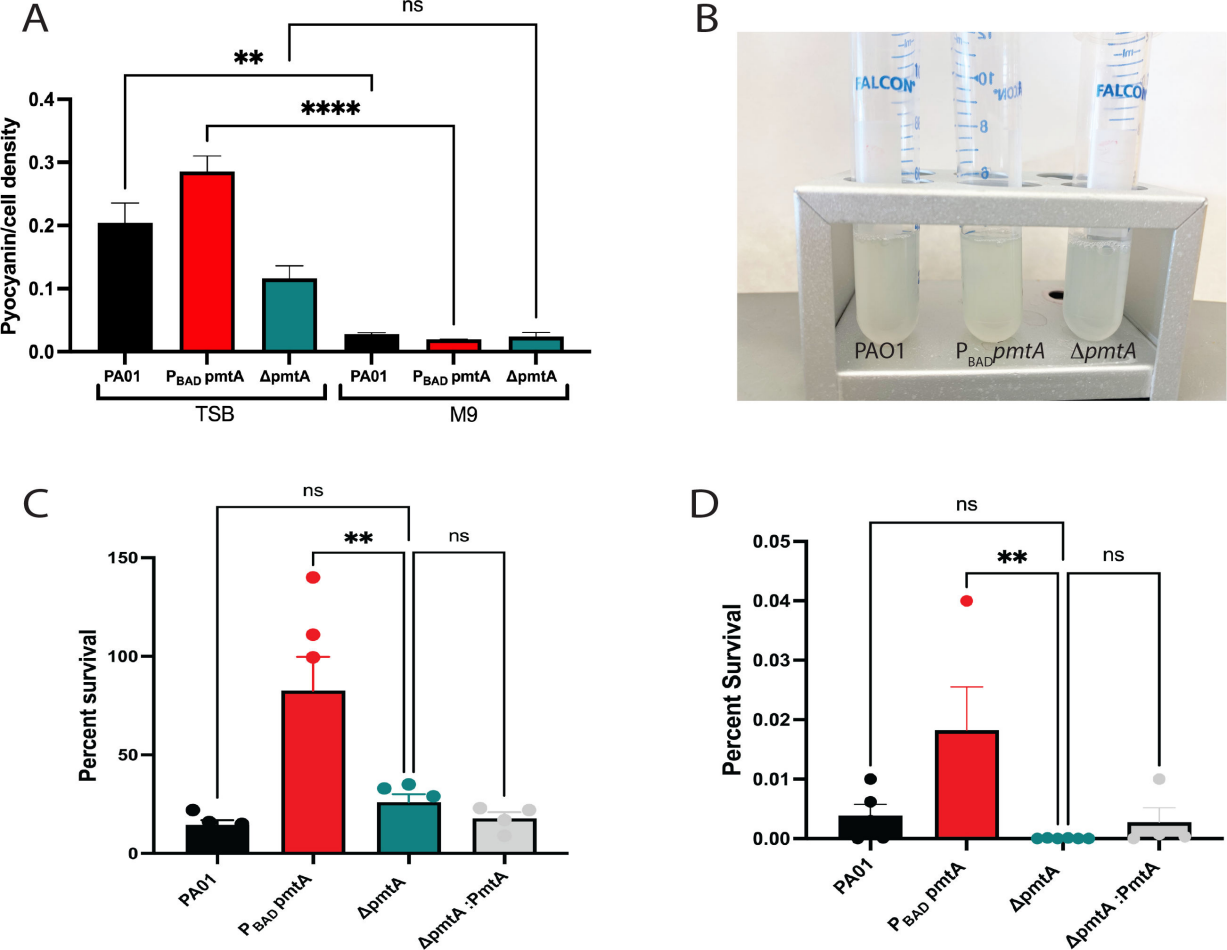
PmtA expression is associated with resistance to oxidative stress *in vitro*. (**A, B**) M9-A media produce reduced pyocyanin levels in *P. aeruginosa* PAO1 strains when compared to TSB-A media. (**A**) Pyocyanin was extracted as previously described (12). Chloroform was added to the supernatant from each strain, forming a blue bottom layer. Subsequently, hydrochloric acid (0.2 M) was added to the layer bringing it to a pH of 2 shifting the color to pink. The absorbance of extracted pyocyanin was measured at OD_520_, and the normalized pyocyanin/cell density was calculated by dividing the pyocyanin at OD_520_ by growth measured at OD_600_. An unpaired *t*-test analysis was performed on these values comparing M9 media to TSB media results (*P < 0.05, **P < 0.01, ***P < 0.001, ****P < 0.0001). (**B**) Image of growth showing no blue green color in M9 media, indicating lack of pyocyanin presence. PmtA expression confers resistance to (**C**) H_2_O_2_ and (**D**) NaOCl. Cultures were grown in M9-A for 18 h, and then, 10^8^ cells were exposed to 40 mM of H_2_0_2_ or 0.3% NaOCl for 90 min. Cultures were then plated on tryptic soy agar (TSA) agar for colony-forming unit (CFU) counting. Percent survival was calculated and is presented as the average of three individual colony replicates (± standard error of the mean). These results are representative of three independent experiments. One-way ANOVA analysis was performed (*P < 0.05, **P < 0.01, ***P < 0.001, ****P < 0.0001).

A variety of ROS are produced by the host immune system as a defense mechanism against invading pathogens. The damage to cells by the oxidation of proteins, lipids, or nucleic acids can be extensive, causing disruptions of metabolic processes, membrane destabilization, and other effects (36). *P. aeruginosa* can employ several mechanisms to respond to ROS stress (36). Given its intrinsic thiol content, PmtA may contribute to antioxidant mechanisms that protect against immune-derived oxidants. Pyocyanin secretion has also been shown to have toxic effects on *P. aeruginosa* secreting these molecules. *P. aeruginosa* offsets these effects by biofilm formation, energy decreases, and by the production of catalase, which can diminish oxidative stress (36). Since pyocyanin production has been shown to be nutrient dependent, we compared pyocyanin levels produced in cultures grown in M9-A to cultures grown in complex media (TSB). Pyocyanin is not secreted at detectable levels in M9-A media compared to TSB (Fig. 3A and B), and so. M9-A was used to minimize the presence of pyocyanin in experiments designed to determine the effects of PmtA on ROS exposure (treating cells with 40 mM H_2_O_2_ or 0.3% NaOCl for 90 mins and calculating the percent survival) (Fig. 3C and D). When we compare P_BAD_*pmtA* to the other PAO1 strains, overexpression of *pmtA* resulted in a higher percent survival when exposed to these two different oxidants, indicating a role for PmtA in relieving oxidative stress. The deletion mutant (*ΔpmtA*) was not significantly different from WT in oxidant sensitivity, which we have also seen in previous studies (20), indicating that when PmtA is not present, *P. aeruginosa* can use other mechanisms to address oxidative stress.

### PmtA provides protection from phagocytosis

**Fig 4:**
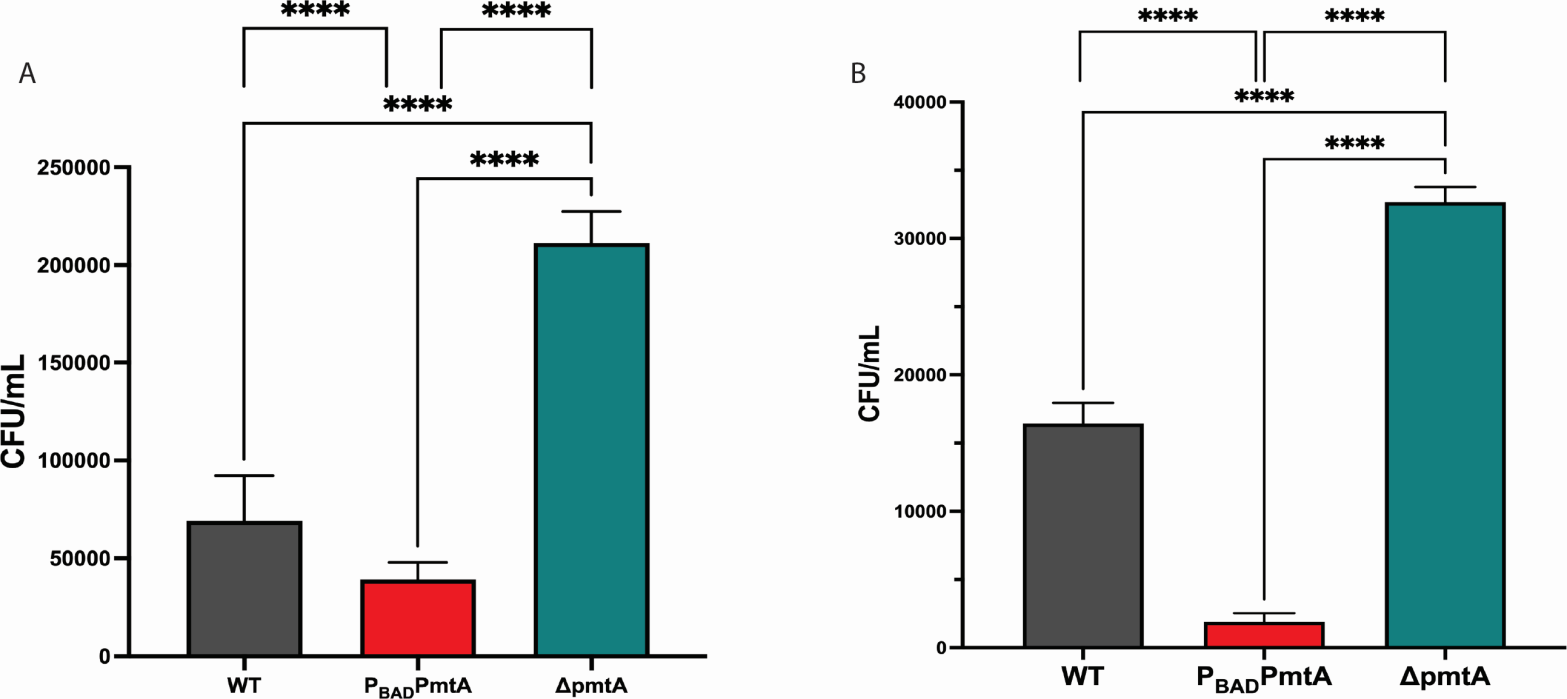
PmtA confers resistance to phagocytosis. (**A**) Phagocytosis assay was performed for 30 min using THP-1 that had been differentiated into a macrophage-like cell using phorbol 12-myristate 13-acetate (PMA). Using gentamicin and washing with PBS, the extracellular populations of Δ*pmtA*, P_BAD_*pmtA*, and/or WT PAO1 strain was eliminated from the coculture. THP-1 cells were then lysed, plated on TSA plates, and CFU/mL was calculated. P_BAD_*pmtA,* when compared to both Δ*pmtA* and WT PAO1, had significantly less CFU/mL recovered indicating a role for PmtA in phagocytosis. (**B**) A 60-min phagocytosis time point was performed as described in (**A**), and again, P_BAD_*pmtA* had significantly less CFU/mL recovered compared to both Δ*pmtA* and WT PAO1 indicating a role for PmtA in phagocytosis. The data are presented as the average of three different colony replicates (± standard error of the mean) and are representative of three independent experiments. One-way ANOVA analysis was performed (A, B), and unpaired *t*-test was performed (**D**) (*P < 0.05, **P < 0.01, ***P < 0.001, ****P < 0.0001).

Since evidence suggests that PmtA plays a role in the management of oxidative stress and in a model of innate virulence (20), we explored the effect of PmtA on phagocytosis in co-culture with a human macrophage cell line (THP-1, ATCC TIB-202). For these experiments, THP-1 cells were treated to differentiate them to an adherent macrophage-like phenotype (37)**,** then were seeded and cultured for 3 days prior to infection (38). Cultures were then infected with ∆*pmtA*, P_BAD_*pmtA*, or WT PAO1 strains (grown in M9-A, washed in phosphate-buffered saline [PBS], and resuspended in MCIM-A) at a multiplicity of infection (MOI) of 10. Live, intracellular bacteria in these infected cultures were determined by plating and counting CFU/mL obtained from cultures that had been antibiotic treated and washed. Unexpectedly, at both time points after infection (30 and 60 min), significantly more live intracellular ∆*pmtA* were observed when compared to both WT and P_BAD_*pmtA* PAO1 strains (Fig. 4A and B). We also observed significantly fewer live intracellular P_BAD_*pmtA* when compared to both ∆*pmtA* and wild-type PAO1 strains at these time points (Fig. 4A and B). These data indicate that the loss of *pmtA* expression results in increased phagocytosis, while overexpressing *pmtA* reduces the frequency of phagocytic engulfment.

### Visualization of PmtA phagocytosis protection

**Fig 5:**
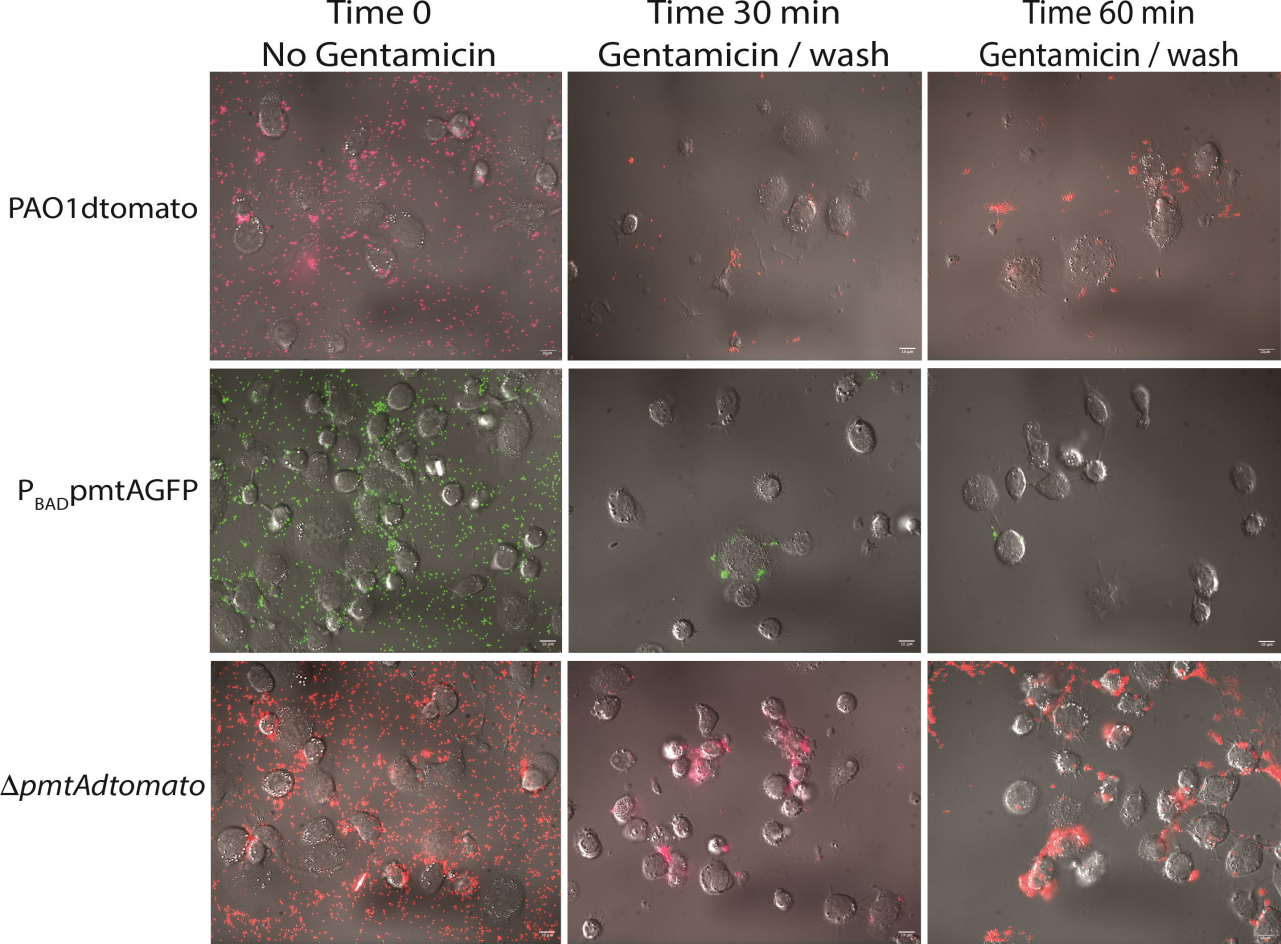
Fluorescent microscopy visualization of PmtA influencing resistance to phagocytosis using fluorescently tagged PAO1 strains. The above phagocytosis assay was performed and imaged using a fluorescence microscope. For scale, a white line on the lower right corner represents 10 µm. At time 0, extracellular bacteria can be visualized. At 30 and 60 min, after the addition of gentamicin and subsequent washing, more Δ*pmtA* can be seen in close proximity to the THP-1 cells when compared to either WT or P_BAD_*pmtA* PAO1.

We visualized phagocytosis of *P. aeruginosa* by tagging each of the PAO1 strains with a fluorescent marker encoded on Tn*7* with a P_BAD_ arabinose-inducible promoter controlling ORFs. The fluorescent proteins dTomato or superfolder GFP (sfGFP) are regulated in Tn7 by constitutively active P_Tac_ promoters (Table 1; Fig. S5). We conjugated the dTomato fluorescent/Tn7 into *P. aeruginosa* ∆*pmtA* and into WT PAO1 strains and verified with PCR (Fig. S5C). We also verified that the *pmtA* mutation was still present in ∆*pmtA:dtomato* after selection of the fluorescent marker transformants and observed no changes in growth profiles (Fig. S5D and E). P_BAD_*pmtA* was labeled with sfGFP fluorescent protein using the same approach. We performed the phagocytosis assay using tagged ∆*pmtA:dtomato*, P_BAD_*pmtA:sfgfp*, and WTPAO1:*dtomato* strains, taking fluorescent images at times 0, 30, and 60 min (Fig. 5). At time 0, extracellular bacteria are visible. Gentamicin was added to coincubations of bacteria with THP-1 at 30 and 60 min, after which the cultures were washed and imaged. Most of the extracellular bacteria were removed by this treatment except for a few that remained attached to the glass bottom of the dish. More ∆*pmtA* bacterial cells were closely associated with THP-1 cells when compared to wild-type and P_BAD_*pmtA* PAO1 strains, indicating a role of *pmtA* in attachment and phagocytosis.

**Fig 6:**
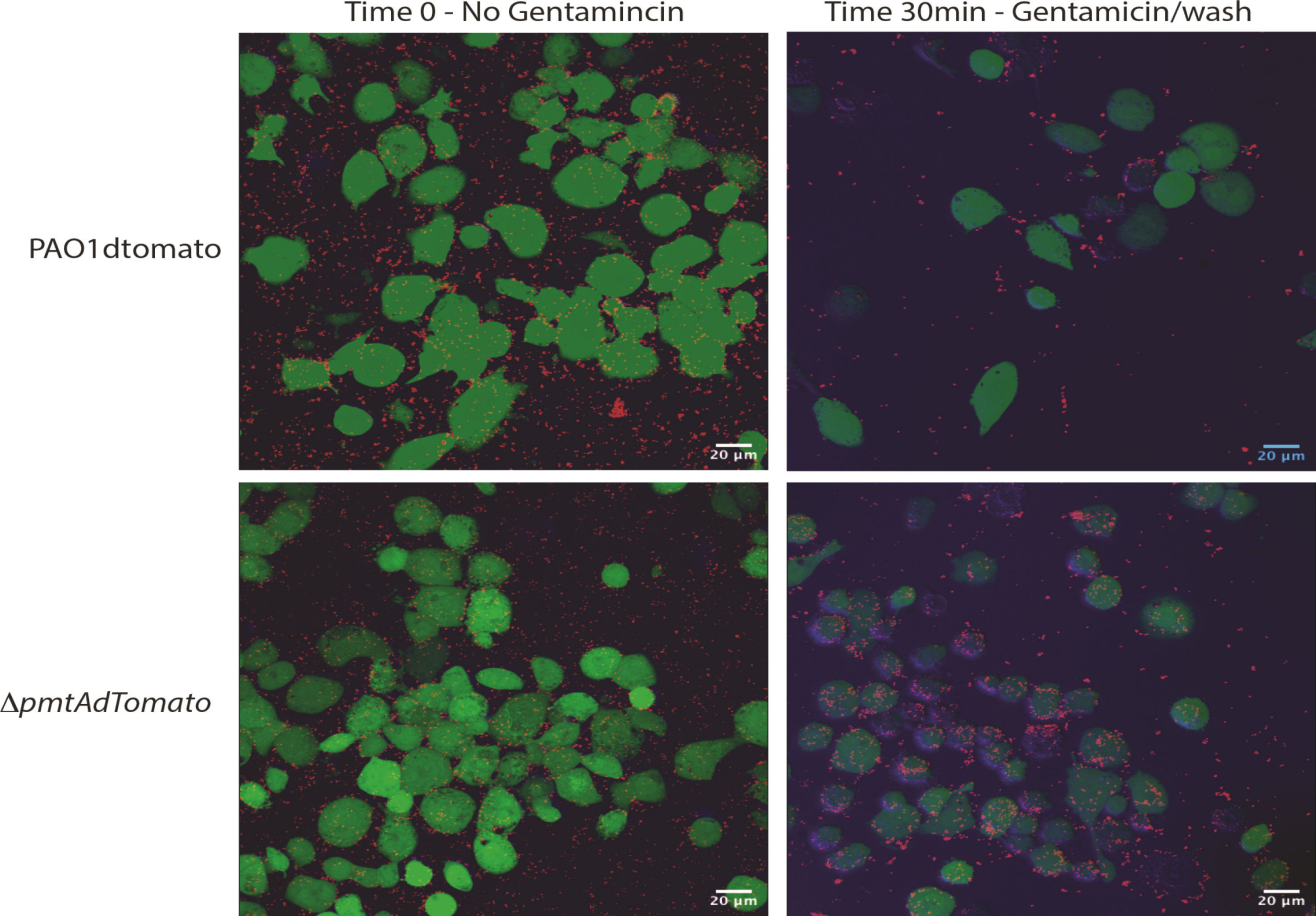
Confocal microscopy of phagocytosis assay using fluorescently tagged WT and Δ*pmtA* PAO1 strains only. The phagocytosis assay was performed for 30 min. Celltracker Green was used to visualize THP-1 cells (which passes through intact cell membranes, allowing visualization of the THP-1 cytoplasm), Z stacks were taken in 0.5-µm steps using a confocal microscope, and a 3D image was generated in ImageJ. At 30 min, after the addition of gentamicin and subsequent washing, more Δ*pmtA* is in close proximity (internalized and attached to the outside) of the THP-1 cells when compared to the WT.

We repeated the phagocytosis assay at the 30-min timepoint and visualized the cultures using confocal microscopy, capturing images across the focal planes that span the THP-1 cell volume (Fig. 6). At 30 min, we again observed more ∆*pmtA* bacterial cells both inside the THP-1 cells and at the cell surface when compared to the wild-type PAO1 strain.

### Anti-PmtA binds to PAO1 surface

**Fig 7:**
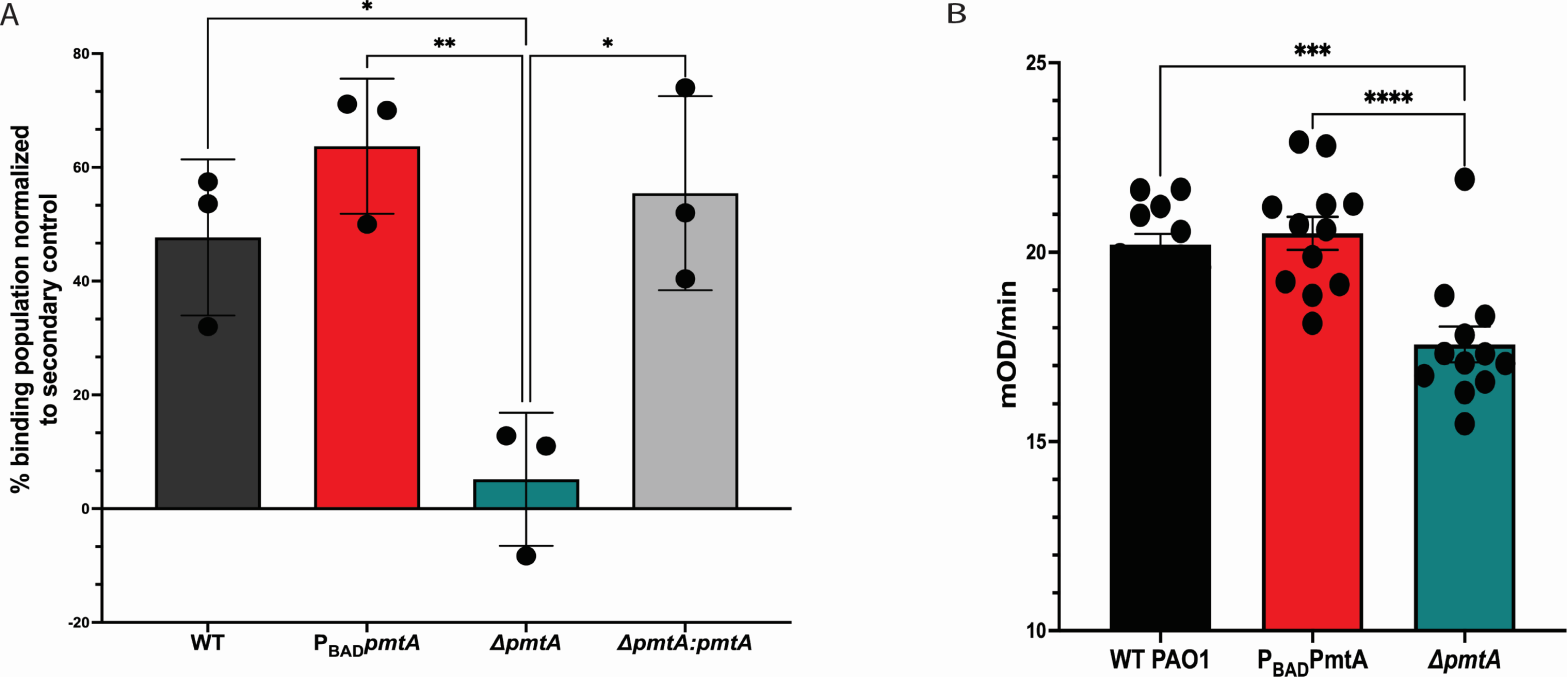
PmtA on PAO1 cell surface. (**A**) Percentage of binding of PAO1 strains incubated with mouse IgG_1_ anti-PmtA with Alexa Fluor 647-labeled goat anti-mouse IgG. Binding was normalized to values for cells stained with Alexa Fluor 647-labeled goat anti-mouse IgG alone. (**B**) Surface binding was tested by ELISA using Immulon 2HB 96-well plates and PAO1 stains. Kinetic OD_405_ values were measured every 30 s for 10 min using a Spectramax plate reader. One-way ANOVA analysis was performed (**P < 0.01 ; ***P < 0.0005 ; ****P < 0.0001).

To identify pathogens for phagocytosis, macrophages use pattern recognition receptors (PRR) to bind microbial-associated molecular patterns (MAMPs) (39). Since we observe more phagocytosis and close association of ∆*pmtA* bacterial cells to THP-1 macrophages, we next examined if PmtA can be found on the surface of WT PAO1. Using a commercially available anti-PmtA monoclonal antibody and fluorescein isothiocyanate (FITC)-labeled goat anti-mouse IgG secondary antibody, we evaluated the binding of anti-PmtA to PAO1 WT strain using fluorescence microscopy (Fig. S6). We observed that PAO1 cells, first incubated with monoclonal mouse IgG1 anti-PmtA followed by a secondary FITC-labeled goat anti-mouse IgG antibody, stained the bacterial cells at a higher level than PAO1 cells exposed to the secondary antibody alone (Fig. S6). We quantified this binding using both flow cytometry and ELISA (40) (Fig. S7; Fig. 7A and B). Anti-PmtA was incubated with each of the three isogenic strains, and then, binding was detected with Alexa Fluor 647-labeled goat anti-mouse IgG. Flow cytometry revealed that monoclonal anti-PmtA antibody binds to PmtA that is available on the surface of WT PAO1 and other *pmtA*-expressing PAO1 strains (Fig. 7A) while having significantly lower binding to ∆*pmtA*. Surface binding of the anti-PmtA mAb can also be detected by ELISA using whole bacteria from PAO1 strains that have been immobilized on the ELISA microwell surface (Fig. 7B). We observed significantly more binding of anti-PmtA on PAO1-expressing strains than on ∆*pmtA*. While monoclonal antibodies are designed to be specific, binding of antibodies to intact, live bacteria has been shown to occur at a lower level owing to nonspecific interactions of antibodies with components of the cell wall, extracellular polysaccharides, and/or extracellular DNA (41, 42).

### PmtA increases persistence within macrophages

**Fig 8:**
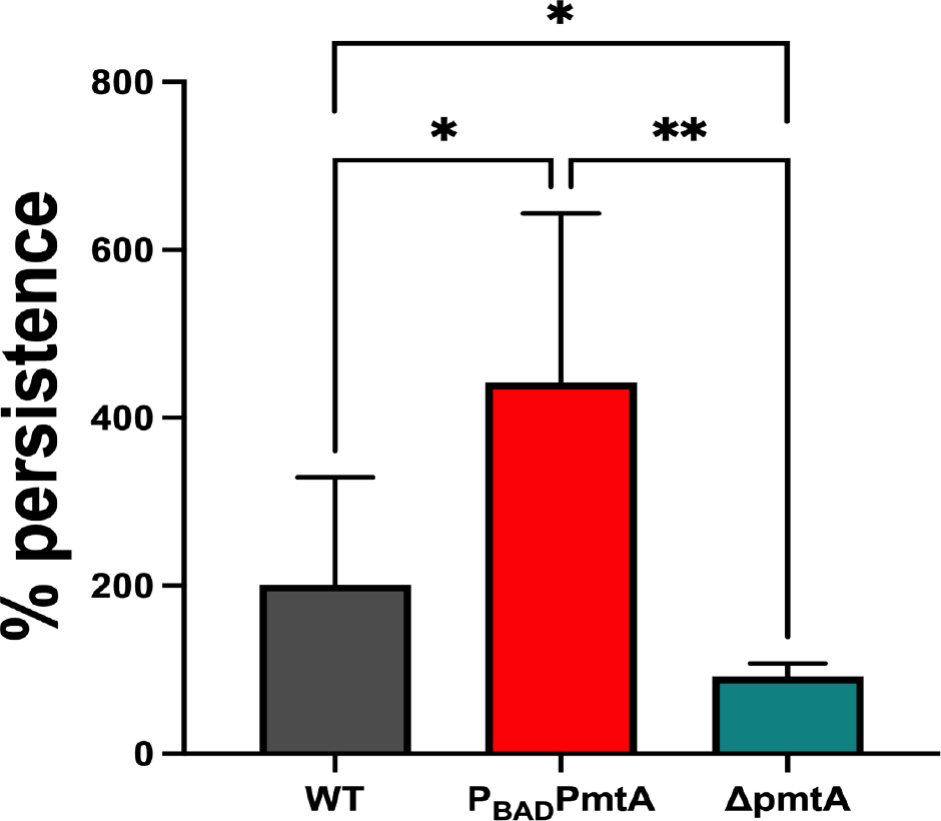
PmtA increases persistence once phagocytosed in THP-1 cells. The phagocytosis assay was performed for 60 min. After the addition of gentamicin and washing the cells, the THP-1 cells were incubated for an additional hour. Then, the cells were lysed, the lysate spread on TSB agar plates, and the persistence rate of recovered bacteria was calculated. Δ*pmtA* had a significantly lower percent persistence when compared to P_BAD_*pmtA* indicating a role for PmtA in intracellular macrophage endurance. Circles represent individual colony replicates (± standard error of the mean) and are representative of three independent experiments. One-way ANOVA analysis (A, B) and unpaired *t*-test were performed (**D**) (*P < 0.05, **P < 0.01, ***P < 0.001, ****P < 0.0001.).

We also evaluated the role of PmtA as a contributor to intracellular survival after phagocytic engulfment. To do this, we repeated the phagocytosis assay as already described, but at 60 min post-infection, we added gentamicin and then continued the THP-1/*P. aeruginosa* co-incubation for another 60 min (Fig. 8). The THP-1 cells were then lysed, and the lysate plated on Tryptic Soy Agar (TSA). We calculated the percent persistence by comparing CFU at 60 min and CFU at 2 h. We found that significantly fewer intracellular ∆*pmtA* bacterial cells were recovered after this 2-h incubation when compared to similarly treated P_BAD_*pmtA* and WT PAO1. These data indicate a role for PmtA in intracellular bacterial persistence that could relate to instances of prolonged or chronic *P. aeruginosa* infection *in vivo*.

## DISCUSSION

These studies show that PmtA enhances bacterial survival under stressful conditions that mimic the innate immune response. ROS plays a central role in the innate immune response against pathogens, and the ability to overcome these host defenses can play a key role in infection. *P. aeruginosa* has many microbial defenses against ROS including antioxidant enzymes such as catalase, superoxide dismutase, peroxidase, and alkyl hydroperoxide reductase (36). The results with the *pmtA*-overexpressing strain *of P. aeruginosa* indicate a role for this protein in the protection from oxidative stress that could be relevant to the expression of pyocyanin, a toxic virulence factor that generates oxidative stress for the host immune cells. Pyocyanin acts by generating host superoxide and H_2_O_2_ through reduction of oxygen, causing damage to the host and nearby bacteria, including self-inflicted damage to *P. aeruginosa* (36). This redox stress, in turn, induces biofilm formation and *de novo* expression of antioxidant enzymes within *P. aeruginosa* and subsequently interferes with bacterial uptake of pyocyanin. Thus, PmtA serving as an antioxidant agent could allow for more pyocyanin production without affecting the infection process. Our previous work also links PmtA to oxidative stress, as restoration of pyocyanin and biofilm defects in the ∆*pmtA* strain was observed with the addition of the antioxidant glutathione (20). Notably, *P. aeruginosa* has an arsenal of mechanisms to respond to oxidative damage, and PmtA might also moderate this stress by possible interactions with these other well-known redox management systems.

Our study also reveals an interesting and unexpected new phenotype; the deletion of *pmtA* causes an increase in phagocytosis by THP-1 macrophages. *P. aeruginosa* is well known for effective phagocytic evasion mechanisms, including the loss of swimming motility to escape phagocytosis (43) and secretion of proteins that can impair the function of macrophages (accomplished by a putative zinc metalloprotease, ImpA) by cleaving multiple macrophage surface proteins (e.g., CD43, CD44, and CD55) (44). We were able to visualize this new phenotype using fluorescent and confocal microscopy and found a greater association of ∆*pmtA* with the THP-1 cells when compared to either WT or P_BAD_*PmtA*. These data indicate that PmtA might play a role in blocking macrophage pattern recognition receptors.

Bacteria have been known to disguise their MAMPs by modifying the PRR ligands and/or other extracellular proteins that they produce (45). While PmtA lacks a signal peptide (as shown by *in silico* evaluation using SignalP software) (46), it does appear to share molecular motifs with other extracellular Gram-negative bacterial proteins using SecretomeP software (47). In this analysis, the PmtA produces a SecP neural network score of 0.662902 (where any score over 0.5 exceeds the threshold as a secreted protein) (47). It is probable that PmtA uses a non-classical secretion pathway or exits during autolysis that occurs during *P. aeruginosa* oxidative stress (48–50).

Both ELISA and flow cytometry can be used to detect PmtA on the surface of *P. aeruginosa*. The path that PmtA uses to arrive at the cell surface remains unknown: PmtA released from cells via non-classical secretion pathways may result in secondary binding to the cell surface from the outside, or PmtA may use as yet undefined cytosolic pathway to reach the bacterial cell surface. Once at the surface, PmtA may block ligands that are detected by PRR-mediated binding used by macrophages to phagocytize certain bacteria (43). Recently, persister cells have been shown to have similar phenotypes to what we see in these studies. Persister cells are variants of normal cells, elicited by stress, that are resistant to antibiotics and provide protection from innate immune response including slow engulfment rates in macrophage phagocytosis and high redox activity upon ceftazidime treatment (10, 51). It is reasonable to speculate on PmtA involvement in persister cell morphology. Future studies will focus on these interactions.

Once inside the macrophage, ∆*pmtA* persistence was significantly reduced when compared to both WT and P_BAD_PmtA. Among the key components of macrophage-associated phagocytic killings are the phagocyte-produced oxidants that enter the phagosome to kill ingested bacteria (52). While there was no significant difference in ∆*pmtA* sensitivity to oxidative stress *in vitro* when compared to WT, P_BAD_PmtA is more resistant to oxidative stress *in vitro*. Our co-culture data show that once ingested by phagocytic cells, PmtA could be an important mechanism to counter oxidative damage, whether this is by modulating other genes important for resistance or acting as an antioxidant alone remains unknown. These findings, along with our previous study (20), indicate an important role for bacterial MT in pathogenicity and suggests PmtA as a possible therapeutic target and diagnostic feature not only for *P. aeruginosa* but for three of the five other ESKAPE pathogens that encode bacterial MT proteins as well.

## MATERIALS AND METHODS

### Reagents

All chemicals and reagents were purchased from Fisher Scientific (Pittsburgh, PA) unless otherwise stated.

### THP-1 cells (human monocytes), media, and supplements

THP-1 cells were cultivated in RPMI-1640 medium (ATCC 30–2001) supplemented with 10% fetal bovine serum, 100 U/mL of penicillin, and 100 mg/mL of streptomycin. The cells were grown in tissue culture flasks, multiwell plates, or glass bottom dishes (WillCo-dish, GWSt-3533) at 37°C and 5% CO_2_. Differentiation into macrophages was induced by treatment with 40 ng/mL of phorbol 12-myristate 13-acetate (PMA) (ThermoFisher Scientific J63916.M resuspended in dimethyl sulfoxide) for 72 h.

### Bacterial strains, media, and growth conditions

*P. aeruginosa strain* PAO1 (53) was used as the wild-type (WT) strain, and all *P. aeruginosa* strains were grown at 37°C in LB containing 10 g of Bacto Tryptone, 5 g of yeast extract, and 10 g of NaCl per liter or TSB or M9 media (arabinose as sole carbon) (20) or on agar-solidified plates or MCIM (RPMI-1640 medium 236.25 mL (ATCC 30–2001), 3.5% FBS , 20 mM HEPES). When bacterial media was supplemented with 1% arabinose, the media was designated as LB-A, TSB-A, M9-A, or MCIM-A). Where a reduced amount of arabinose was added, the concentration was noted. For agar plates, 15 g/L of Bacto Agar (BA) was added. BA contained 44 g of Columbia BA base per liter. For each assay, overnight cultures were established from a single colony. All relevant bacterial strains and plasmids are described in Table 1. Antibiotic selection for conjugations was performed with 1.5 mg/mL of trimethoprim and 25 mg/mL of spectinomycin as previously described (26). Plasmids were maintained in *Escherichia* coli using 100 mg/mL of trimethoprim, 100 mg/mL of ampicillin, and 100 mg/mL of kanamycin.

### Generation of overexpressing *pmtA P. aeruginosa* strain PAO1 and fluorescently tagged PAO1 strains

The full-length of the *pmtA* gene (258 bp) was PCR amplified from *P. aeruginosa* strain PAO1 genomic DNA (gDNA) using the primer pairs pmtAFEcoR1 (TACCCATGGGATCTGATAAGAATTCATGAACAGCGAAACCTGTGC) and pmtARHindIII (GTACCGGGCCCGCGGCCGCAAGCTTGCTCCTCAGGGCGAGATC). The PCR mixture contained 2× Phusion High-Fidelity PCR Master Mix with HF Buffer (New England Biolabs [NEB]; Cat.#: M0531S/L), 10 mM of pmtAF, 10 mM of pmtAR, and 25 ng/mL of gDNA in a final volume of 50 mL. The amplification conditions were as follows: (i) 4 min at 98°C and (ii) 30 cycles of 25 s at 98°C, 30 s at 63°C; and 60 s at 72°C; and (iii) 10 min at 72°C. Simultaneously, pTJ19 was linearized using EcoRI-HR (NEB R3101T) and HindIII-HF (NEB R3104T) following manufacturer’s suggested conditions. Amplification fragments were assembled using Gibson assembly (NEB E2611S) and transformed into NEB5-alpha (NEB C2987H) competent *E. coli* cells following manufacturer conditions yielding pBADPmtA. The suicide delivery vector (pBADPmtA) was verified for correct fragment orientation by sequencing using Plasmidsaurus.com ( Fig. S1). A quadriparental mating was performed to conjugally transfer the Tn*7* with a P_BAD_ arabinose-inducible promoter in front of the *pmtA* gene from pBADPmtA into the *glmS* intergenic region of PAO1, yielding strain P_BAD_*pmtA*. The insertion of the Tn7 downstream of *glmS* was verified as previously described (20, 26, 29, 54) (Fig. S1).

PAO1 strains were also fluorescently tagged by generated a Tn*7* with trimethoprim plus ORFs encoding the fluorescent proteins dTomato or sfGFP. Expression scaffolds and Tac promoters were PCR amplified from pTW41552 and pTW416 (35) using the following primer sets, respectively, and as described above: GFPpmtAF (GCCTTCGCGAGGTACCAAGGGCAGATTGTGTCGACC) and GFPpmtAR (TTGCGGCCGCGGGCCCGCTAATTCGATCATGCATGAGCTCAC) dtomatoR (ACCGGGCCCGCGGCCGCAAGCTTGCATGAGCTCACTAAATTCTTGACAATTAAT) and dtomatoF (TCGACCTGCAGGCATGCAAGCTTGACCATGTGGTCACGCTTTTCG). For the over-expressing PAO1 strain using a GFP tag, pBADPmtA was linearized using restriction enzymes ApaI (NEB R0114S) and KpnI (R3142S) following manufacturer conditions. For both WT PAO1 and D*pmtA* using the dTomato tag, pTJ19 was linearized using HindIII-HF (NEB R3104T) following manufacturer’s recommended conditions. Amplification fragments were assembled using Gibson assembly (NEB E2611S) and transformed into NEB5-alpha (NEB C2987H) competent *E. coli* cells following manufacturer’s conditions yielding the following suicide delivery vectors, pPBADpmtAGFP, and pdTomatoPBADTpTn7 (Fig. 5S). These suicide delivery vectors were verified for correct fragment orientation by sequencing using Plasmidsaurus.com (Fig. S5). A quadriparental mating was performed as described above in WT PAO1 and D*pmtA* yielding; PAO1dtomato, D*pmtA*dtomato, and P_BAD_*pmtA*GFP. The insertion of the Tn7 downstream of *glmS* was verified as previously described (20, 26, 28, 29, 33, 54) (Fig. S5C). To reverify that D*pmtA*dtomato maintained the clean deletion, *pmtA* was PCR amplified as previously described from all new strains (Fig. S5D lane 3) (20).

### Pyocyanin quantification

*P. aeruginosa* strains were grown for 30 h in triplicate, and 1 mL of cell-free supernatants were harvested. Pyocyanin was measured as previously described (20).

### Biofilm formation assay

Biofilm formation was quantified using a previously described microtiter plate assay (20).

### Congo red colony morphology assay

Colony morphology assays were performed as previously described (55–57), with the following modifications: solid media was composed of 1% tryptone, 1% agar, 40 mg/mL of Congo red (Fisher Scientific, S70401-1), and 20 mg/mL of Brilliant Blue Coomassie (Fisher Scientific, BP101-25), which were added into 500 mL of deionized water and autoclaved for 20 min before pouring plates. Overnight cultures of D*pmtA*, P_BAD_*pmtA*, and WT PAO1 strains were grown for 24 h in M9 media. Five microliters of overnight cultures was spotted on colony morphology assay media and incubated at 25°C, >95% humidity for 5 days. Images of the pink centers from the biofilm were analyzed using ImageJ software (58).

### Oxidative *in vitro* exposure assay

Overnight cultures of D*pmtA*, P_BAD_*pmtA*, and WT PAO1 strains were grown for 24 h in M9 media. From each strain, 10^8^ cells were added to 1 mL of fresh M9 media, and starting the CFU/mL was verified by serial dilutions and plating. Cells were exposed to either 40 mM of H_2_O_2_ or 0.3% NAOCl for 90 min at 37°C. Then, cultures were serial diluted and plated for end point CFU/mL determination. Percent survival was calculated using end point CFU/mL/starting CFU/mL.

### Quantitative PCR (qPCR) P_BAD_ arabinose-inducible promoter verification

Overnight cultures of D*pmtA*, P_BAD_*pmtA*, and WT PAO1 strains were grown for 24 h in M9-A, TSB-A, LB-A, or MCIM-A. Total RNA was extracted using manufacturer’s RNA extraction protocol from MasterPure DNA and RNA purification kit (Biosearch technologies). RNA was converted to cDNA with an iScript cDNA synthesis kit (Bio-Rad, 1708890). Gene-specific primers to *pmtA* and the housekeeping ribosomal gene (RNA polymerase, subunit alpha) were used as previously described for qPCR with the following modifications (12). All reactions were setup in 20-mL volume including the following: iTaq Universal SYBR Green Supermix (50% of reaction) (Bio-rad 1725120) 10 mM forward and reverse primers (15% of reaction), nuclease-free H_2_O (10% of reaction), and 100 ng of cDNA. Negative controls with no template were prepared and tested with each set of reactions. Reactions were amplified in triplicate using a CFX96 Real-Time Thermocycler (Bio-Rad) following the thermal cycle protocol as recommended by the manufacturer. The relative gene expression for *pmtA* was calculated using the cycle threshold (Ct) value compared to RNA polymerase, with the alpha subunit (*RpoA*) gene as the internal reference standard. Fold changes were expressed as 2^−ΔΔCt^ values.

### Phagocytosis assay

THP-1 cells were differentiated using PMA (38) and seeded in 24-well plates (1 × 10^5^ cells/well), 3 days before infection with PAO1 strains at an MOI of 10. Phagocytosis assays were carried out by growing PAO1 strains in M9-A media for 24 h. A total of 10^8^ cells were washed 2 times with PBS and serial diluted in infection media containing 1% arabinose. Cells were then incubated for 30 or 60 min at 37°C in 5% CO_2_ in air. Gentamicin (300 mg/mL) was then added to each well, and an additional 30 min of incubation was performed. Afterward, the cells were gently washed three times with PBS, and macrophages were detached with 2 mM of EDTA and lysed with 0.1% Triton-X 100. The lysed cells were serially diluted and plated on TSA, and CFU/mL was calculated.

For imaging, the differentiated THP-1 cells (1 × 10^6^) were plated 3 days before infection on Bioptechs 30-mm glass bottom culture dishes. Cells were exposed to PAO1 strains at an MOI of 10 and gentamicin treated/washed as described above. Cells were imaged on a Zeiss Axiovert 100 fluorescence microscope with a ×63 oil immersion objective or on Nikon AXR confocal with ×66 oil immersion objective. For confocal images, Celltracker Green (Invitrogen, catalog no. C7025) was used to visualize THP-1 cells. Images were acquired using an Imaging QIClick camera and processed using ImageJ (58).

### Immunostaining with anti-pmtA

PAO1 was grown overnight in M9 media, and 10^8^ cells were washed twice with PBS. The bacteria were resuspended in 20 mL of PBS. Bacteria were then heat fixed onto the slide, and 20 mM of mouse monoclonal IgG_1_ anti-PmtA (StressMarq Biosciences Inc., SMC-553, clone 8D8) in 50 mL of PBS was incubated on the fixed cells for 1 h at RT. The slide was washed 3× with 1000 mL of PBS. FITC-labeled goat anti-mouse IgG secondary antibody (SouthernBiotech, 1030-02) was diluted 1:400 in PBS, added to the slide and incubated 1 h at room temperature (RT) in the dark. The slide was washed 3× with 1,000 mL of PBS, and then, cells were imaged on a Zeiss Axiovert 100 fluorescence microscope with a ×63 oil immersion objective or on Nikon AXR confocal with a ×66 oil immersion objective. Images were acquired using an Imaging QIClick camera and processed using ImageJ (58).

### Flow cytometry PmtA binding assay

Flow cytometry binding assay was performed as previously described with the following modifications (40). *P. aeruginosa* strains were grown overnight in M9 media, and 10^7^ bacterial cells were washed twice with 1 mL of PBS. Cells were then resuspended in 100 mL with 1.67 × 10^−5^ M of mouse anti-PmtA. Bacteria–antibody mixtures were incubated for 1 h at 4°C then spun down for 5 min at 15,000 × *g*. Bacterial cells were then washed with 1 mL of PBS. Bacteria were then resuspended, pelleted, and then resuspended in 1:400 Alexa Fluor 647-labeled goat anti-mouse IgG for 1 h at 4°C in the dark. Bacteria were then spun down for 5 min at 15,000 *× g* and washed with 1 mL of PBS. Cells were then fixed for 1 h at 4°C with 4% (vol/vol) paraformaldehyde. After fixation, cells were centrifuged at 15,000 × *g* for 5 min and resuspended in 300 mL of PBS. Samples were analyzed using flow cytometry on a BD Fortessa. Bacteria were gated using their forward and side scatter profile and analyzed for Alexa-Fluor 647 intensity.

### PmtA surface-binding characterization using ELISA

PmtA accessibility on the surface of intact bacterial cells was tested with monoclonal anti-PmtA binding using ELISA in Immulon 2HB 96-well plates as previously described with the following modifications (40). *P. aeruginosa* strains were grown overnight in M9 media, and 10^8^ bacterial cells were washed twice with 1 mL of PBS. Microtiter plates then were coated with 100 mL of 5 × 10^7^ CFU prewashed cells resuspended in PBS and incubated overnight at 4°C. Plates were then washed and blocked with 2% bovine serum albumin overnight at 4°C. Following another wash step, the plate was then incubated with 5 mg/mL of anti-PmtA for 1.5 h at RT, subsequently washed, and incubated with goat anti-mouse Ig-AP (SouthernBiotech, 1010-04) for 1.5 h at RT. After the final wash, 1 mg/mL of p-nitrophenyl phosphate substrate was added to the plates, and optical density values at 405 nm were measured every 30 s for 10 min using a Spectramax (Molecular Devices) plate reader.

### *P. aeruginosa* intracellular persistence macrophage assay

THP-1 cells were differentiated using PMA and seeded in 24-well plates (1 × 10^5^ cells/well) 3 days before infection with PAO1 strains at an MOI of 10. Phagocytosis assay was performed as described above, and infected cells were incubated for 60 min at 37°C in 5% CO_2_. Then, 300 mg/mL of gentamicin was added to each well. For selected wells, an additional 30 min of incubation was performed. Cells were gently washed three times with PBS, and macrophages were detached with 2 mM of EDTA and lysed with 0.1% Triton-X 100 at time zero (CFUt_0_). In the remaining wells, gentamicin (300 mg/mL) was added and then incubated for an addition 60 min (CFUt_60_). There was no difference in gentamicin sensitivity between the different *P. aeruginosa* strains. At the end of the incubation, the cells were gently washed three times with PBS, and macrophages were detached with 2 mM of EDTA and lysed with 0.1% Triton-X 100. The lysed cells were serially diluted and plated on TSA, and CFU/mL and percent persistence (*P* = CFU_t60_/CFU_t0_ × 100) was calculated.

## SUPPLEMENTAL MATERIAL

Figure S1 msphere.00210-24-s0001.tif Plasmid map and PCR amplification.

Figure S2 msphere.00210-24-s0002.tiff Cultures grown in TSB, with either 0.5% arabinose or 1% arabinose.

Figure S3 msphere.00210-24-s0003.tiff Cultures grown in LB, with 0.5% arabinose and 1% arabinose.

Figure S4 msphere.00210-24-s0004.tiff Cultures grown in M9 media.

Figure S5 msphere.00210-24-s0005.png Plasmid maps and PCR amplification.

Figure S6 msphere.00210-24-s0006.png Fixed cells stained with only FITC-labeled goat anti-mouse IgG or both mouse anti-PmtA and FITC-labeled goat anti-mouse IgG secondary antibody.

Figure S7 msphere.00210-24-s0007.png Flow cytometry detection of anti-PmtA binding to PAO1 and ∆pmtA.

Legends msphere.00210-24-s0008.docx Supplemental figure legends.

ASM does not own the copyrights to Supplemental Material that may be linked to, or accessed through, an article. The authors have granted ASM a non-exclusive, world-wide license to publish the Supplemental Material files. Please contact the corresponding author directly for reuse.
