# Supplementary figures and images for: Bacterial metallothionein, PmtA, a novel stress protein found on the bacterial surface of Pseudomonas aeruginosa and involved in management of oxidative stress and phagocytosis
Source: mSphere. 2024 May 7;9(5):e00210-24. doi: 10.1128/msphere.00210-24 (PMC11237414; doi:10.1128/msphere.00210-24)

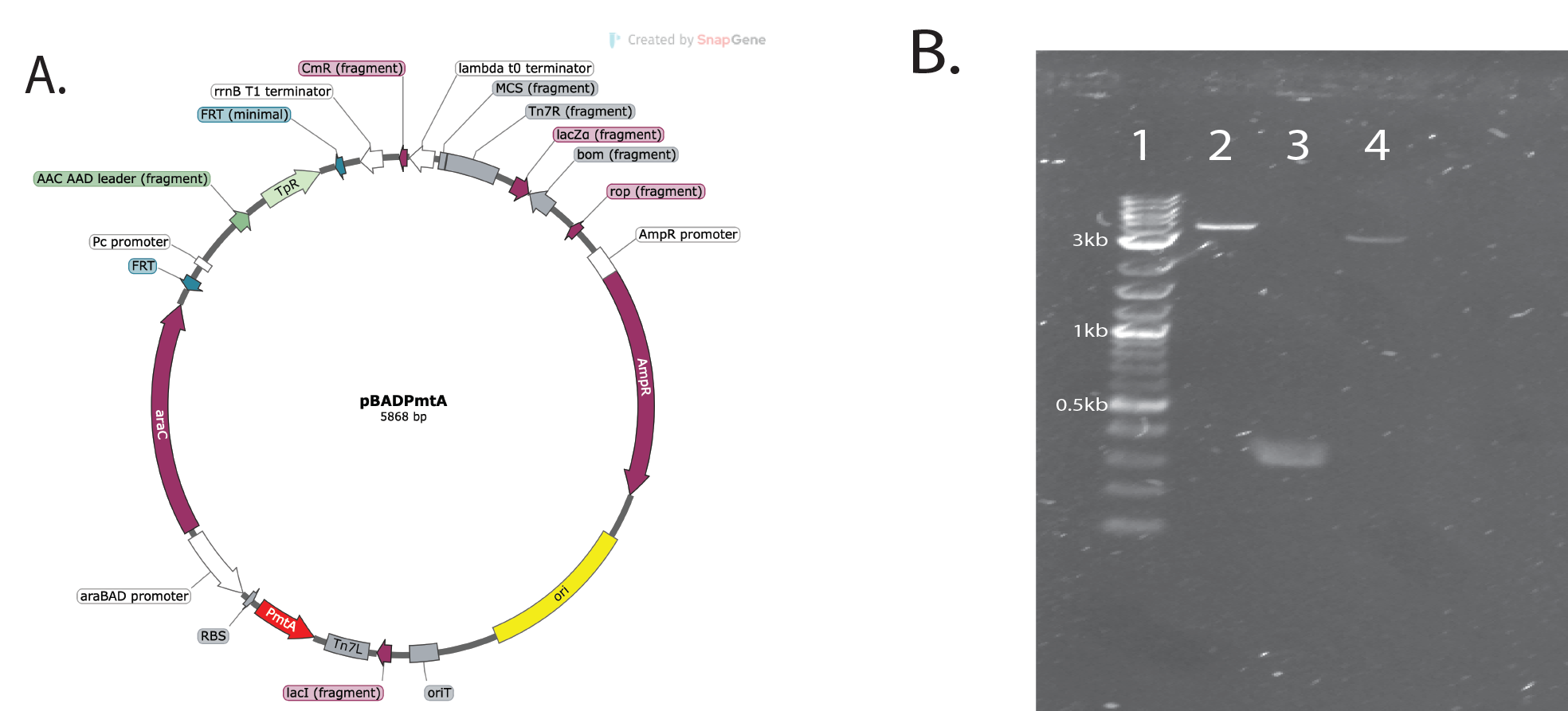

Supplement: Figure S1 — Plasmid map and PCR amplification. [file msphere.00210-24-s0001.tif]

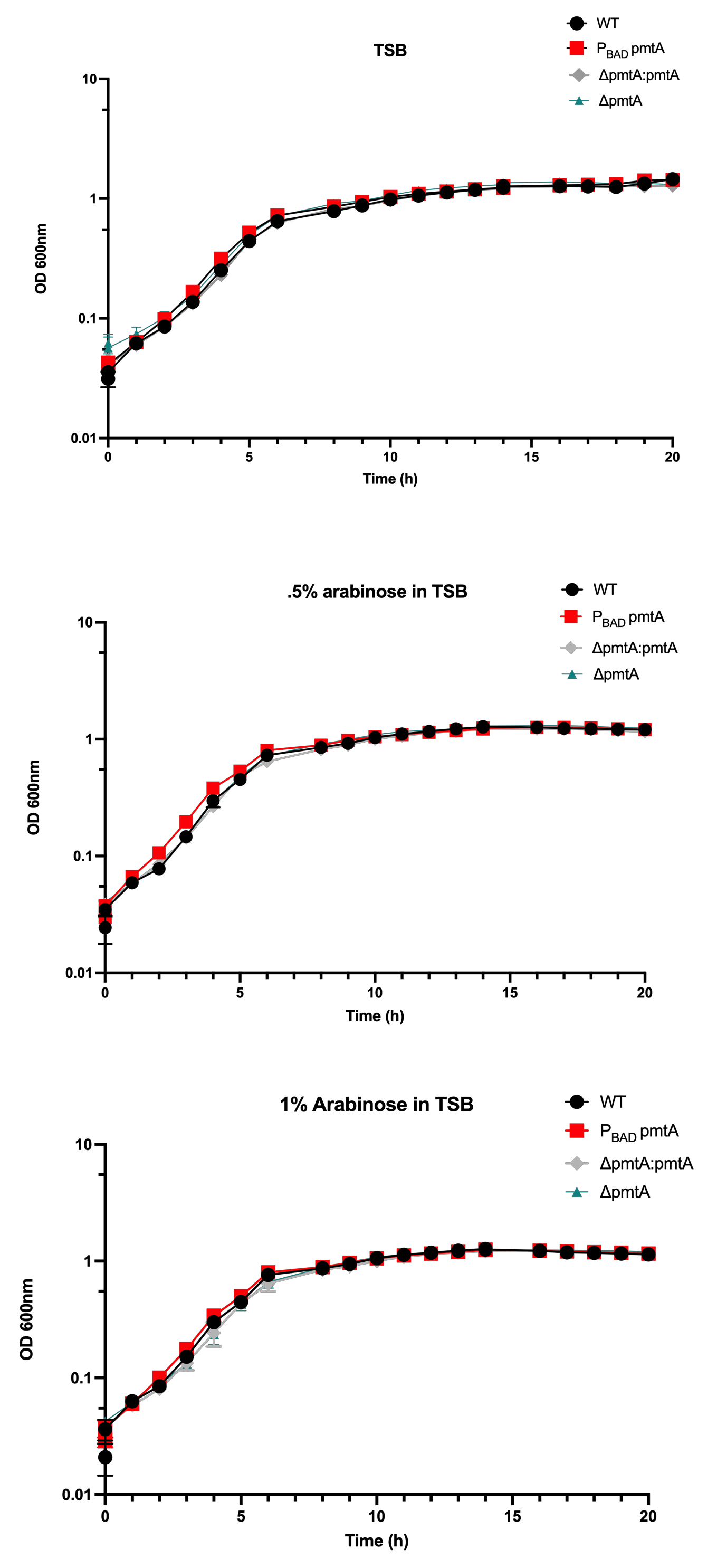

Supplement: Figure S2 — Cultures grown in TSB, with either 0.5% arabinose or 1% arabinose. [file msphere.00210-24-s0002.tiff]

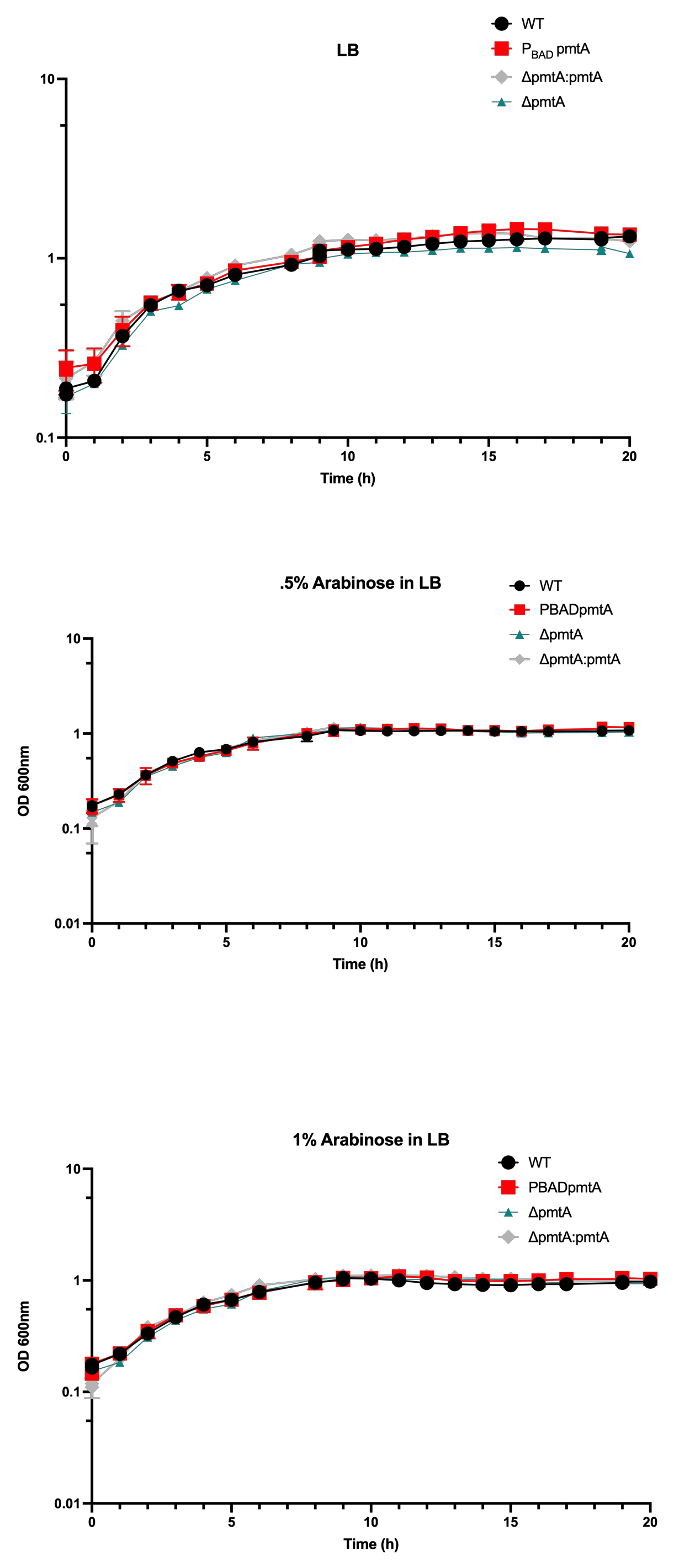

Supplement: Figure S3 — Cultures grown in LB, with 0.5% arabinose and 1% arabinose. [file msphere.00210-24-s0003.tiff]

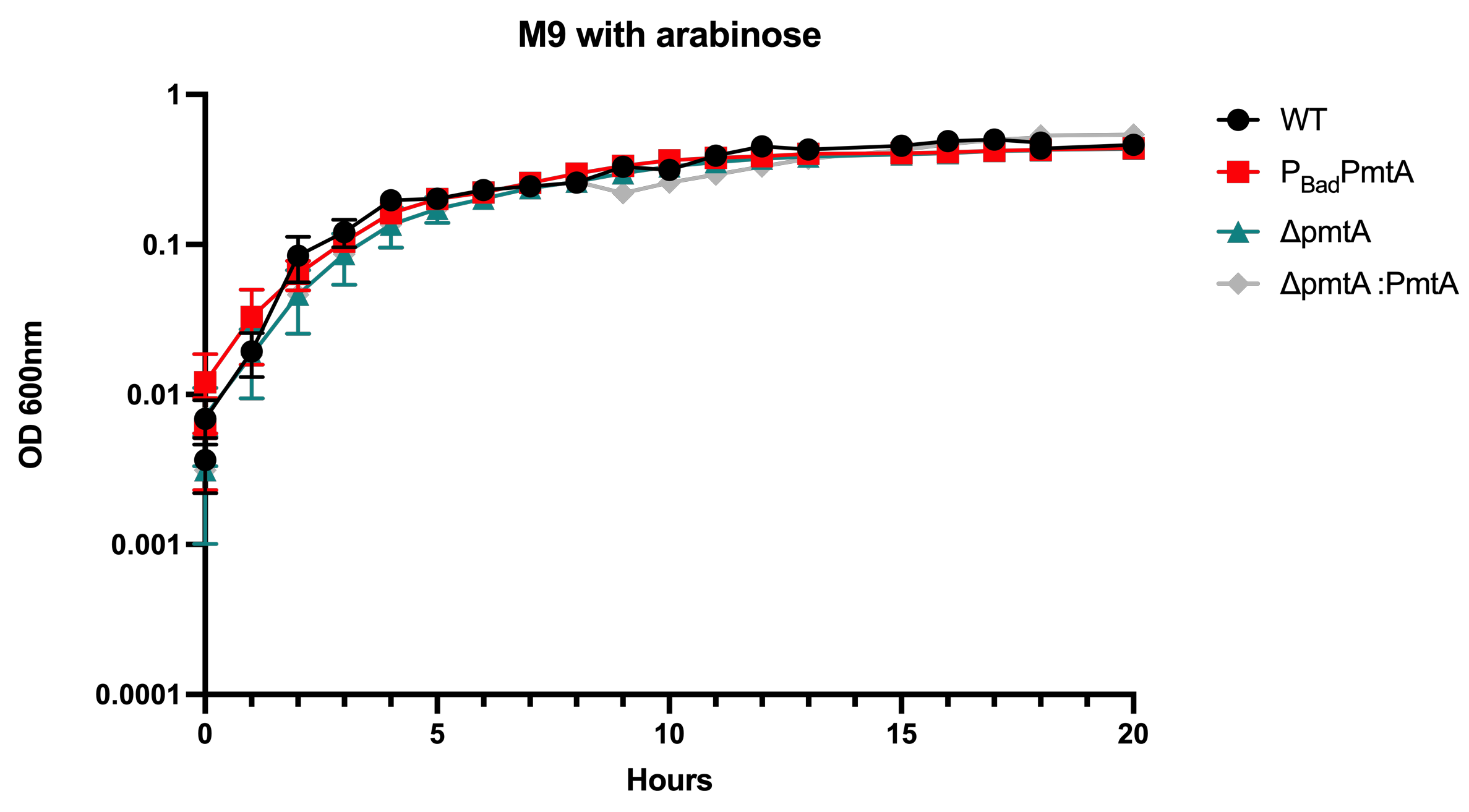

Supplement: Figure S4 — Cultures grown in M9 media. [file msphere.00210-24-s0004.tiff]

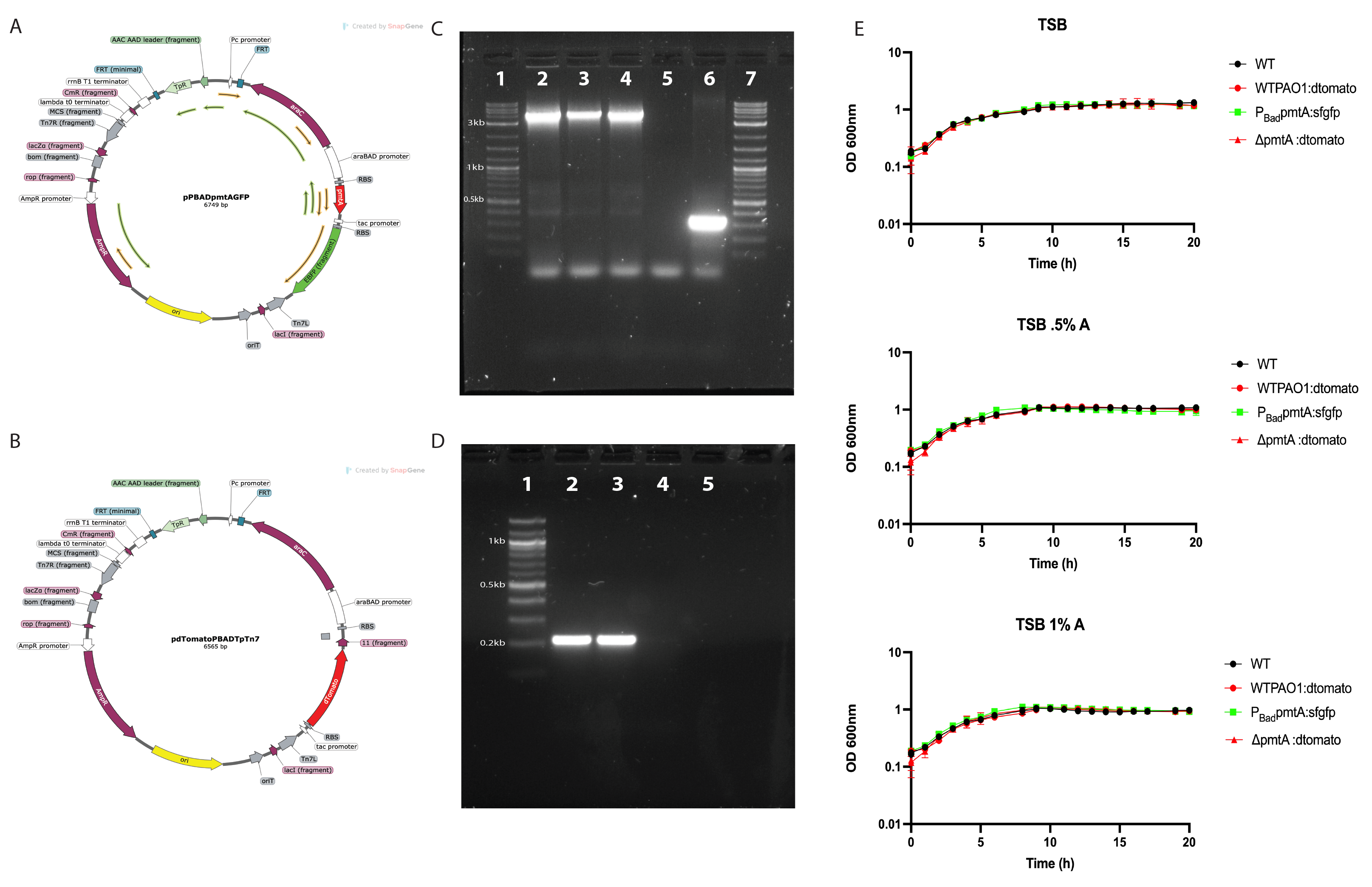

Supplement: Figure S5 — Plasmid maps and PCR amplification. [file msphere.00210-24-s0005.png]

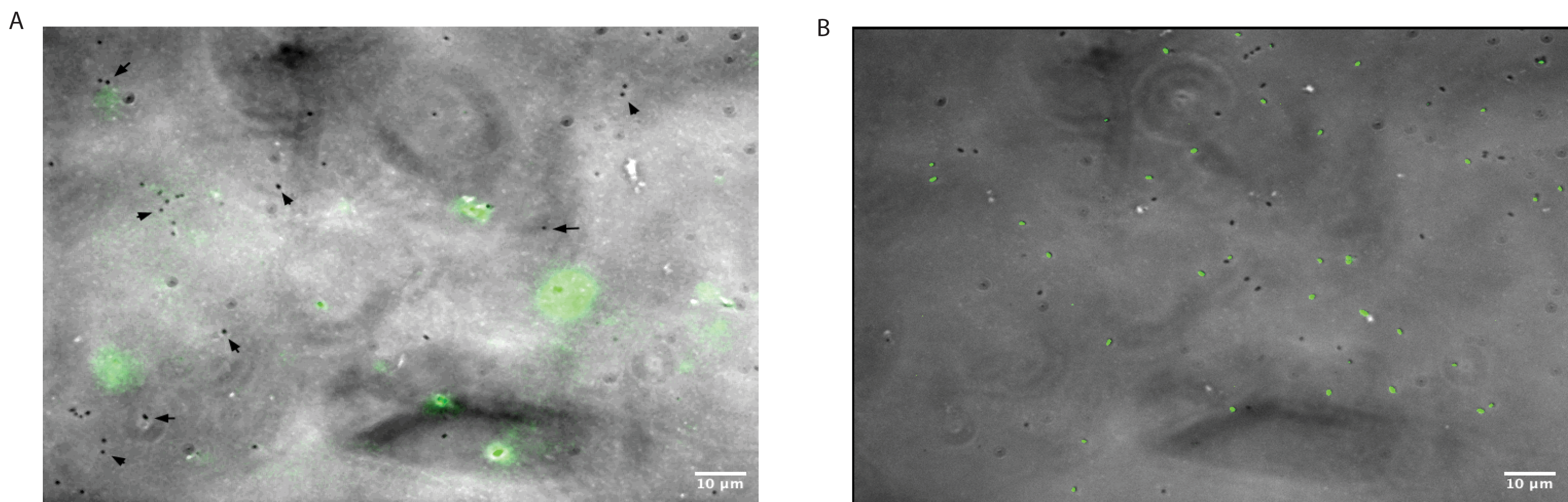

Supplement: Figure S6 — Fixed cells stained with only FITC-labeled goat anti-mouse IgG or both mouse anti-PmtA and FITC-labeled goat anti-mouse IgG secondary antibody. [file msphere.00210-24-s0006.png]

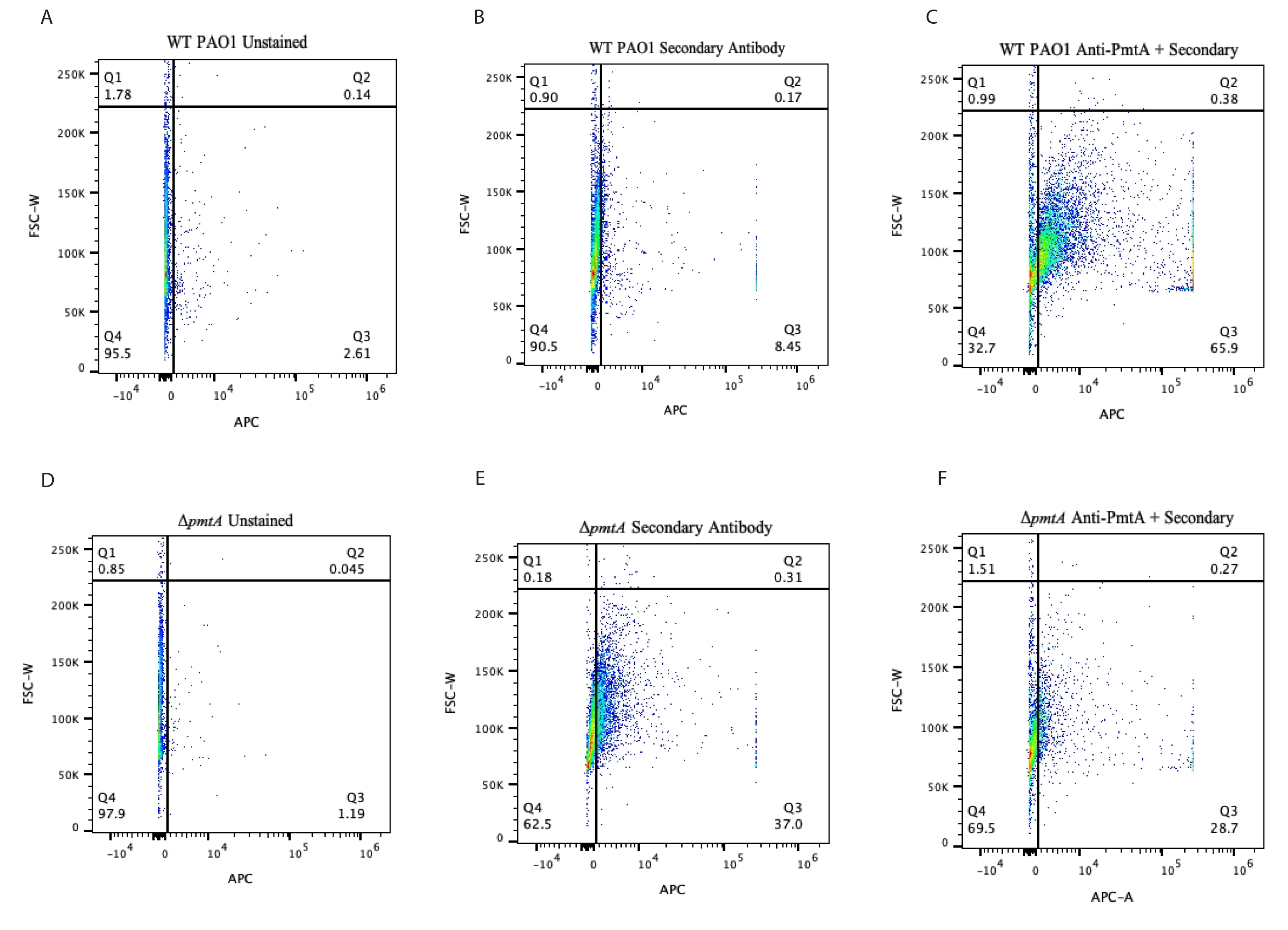

Supplement: Figure S7 — Flow cytometry detection of anti-PmtA binding to PAO1 and ∆pmtA. [file msphere.00210-24-s0007.png]
