# Supplementary material for: Bacterial metallothionein, PmtA, a novel stress protein found on the bacterial surface of Pseudomonas aeruginosa and involved in management of oxidative stress and phagocytosis
Source: mSphere. 2024 May 7;9(5):e00210-24. doi: 10.1128/msphere.00210-24 (PMC11237414; doi:10.1128/msphere.00210-24)
Supplement: Legends — Supplemental figure legends. [file msphere.00210-24-s0008.docx]

**Supplemental Figure Legend**

**Figure 1S generation of P_BAD_*pmtA***

(A) Plasmid map generated from Plasmidsaurus.com sequencing showing *pmtA* inserted into pTJ1 generating pBADpmtA. (B) PCR amplification of the *glmS* region from *P*_BAD_*pmtA* showing a 3.2kb band (lane 2), PAO1 showing 300bp band (lane 3), and $\Delta$*pmtA:pmtA* showing 3kb band (lane 4). The band shift in *P*_BAD_*pmtA* and $\Delta$*pmtA:pmtA* when compared to PAO1 indicates the Tn7 insertion. Lane 1 shows migration of a 1kb DNA ladder.

**Figure 2S P_BAD_*pmtA*** **growth curves compared to PAO1 Strains in TSB**

Cultures were grown in a 24 well plate with TSB, with either 0.5% arabinose or 1% arabinose. Cultures were monitored over 15 h at 37◦C with 5 s shaking every 1 h in a Spectramax microplate incubator/reader at OD_600_.

**Figure 3S P_BAD_*pmtA*** **growth curves compared to PAO1 Strains in LB**

Cultures were grown in a 24 well plate with LB, 0.5% arabinose and 1% arabinose. Cultures were monitored over 15 h at 37◦C with 5 s shaking every 1 h in a Spectramax microplate incubator/reader at OD_600_.

**Figure 4S P_BAD_*pmtA*** **growth curves compared to PAO1 Strains in M9**

Cultures were grown in a 24 well plate with M9 Media and monitored over 15 h at 37◦C with 5 s shaking every 1 h in a Spectramax microplate reader at OD_600_. The data are presented as the average of three biological replicates.

**Figure 5S Generation of fluorescently tagged PAO1 strains and validation.** (A) Plasmid map generated from Plasmidsaurus.com sequencing showing EBFP (Enhanced blue variant of GFP) inserted into pBADpmtA resulting in pBADpmtAGFP. (B) Plasmid map generated from Plasmidsaurus.com sequencing showing dtomato inserted into pTJ1 generating pdTomatoPBADTpTn7. (C) PCR amplification of the *glmS* region from WTPAO1:*dtomato* showing a 3.8 kb band (lane 2), P_BAD_ *pmtA:sfgfp* a showing 4 kb band (lane 3), ∆*pmtA*:*dtomato* showing a 3.8 kb band (lane 4) and PAO1 showing a 300 bp band (lane 6). Lane 5 is the negative control and lane 1 and 7 are the 1 kb ladder (NEB). The band shift in WTPAO1:*dtomato*, P_BAD_ *pmtA:sfgfp* and ∆*pmtA*:*dtomato* when compared to PAO1 represents the Tn*7* insertion. (D) PCR amplification of *pmtA* region from WTPAO1:*dtomato* (lane 2), P_BAD_ *pmtA:sfgfp* (lane 3) and ∆*pmtA*:*dtomato* (lane 4). The absences of a band in ∆*pmtA*:*dtomato* indicates the clean deletion mutant is still present after Tn7 selection (∆*pmtA*:*dtomato*).

**Figure 6S Visualization of Anti-PmtA binding to PAO1 cells.**

(A) Fixed cells stained only with FITC-labeled goat anti-mouse IgG secondary antibody visualized by fluorescence microscopy shows minimal binding, black arrows show individual bacterium. (B) Fixed cells stained with both mouse anti-PmtA and FITC-labeled goat anti-mouse IgG secondary antibody shows more binding.

**Figure 7S Flow detection of anti-PmtA**. Representative plots by flow cytometry detection of anti-PmtA binding to PAO1 (panels A,B,C) and ∆*pmtA* (Panels D,E,F) using Alexa Fluor^TM^ 647 labeled goat anti-mouse IgG (panels C,F). Controls are unstained cells (panels A.D) and cells stained with Alexa Fluor^TM^ 647 labeled goat anti-mouse IgG alone (panels B,E).
